# Systematic review of unveiling the potential of AI using machine learning and deep learning methods in neurodegenerative diseases

**DOI:** 10.7717/peerj-cs.3860

**Published:** 2026-06-26

**Authors:** S. Mohanraj, Sujatha Radhakrishnan

**Affiliations:** School of Computer Science Engineering and Information Systems, Vellore Institute of Technology, Vellore, Tamil Nadu, India

**Keywords:** Neurodegenerative diseases (NDD), Huntington’s disease (HD), Alzheimer’s disease (AD), Parkinson’s disease (PD), Multimodal data

## Abstract

**Background:**

Neurodegenerative diseases (NDDs) are becoming a major worldwide issue, especially for the elderly because they are incurable and permanent. It is extremely difficult to provide any medication to people suffering from NDDs. Comprehending essential processes of NDDs are essential to establishing alternative strategies that can increase survival rates in patients. This review is an effort to provide insight into NDDs, innovative therapeutic methods along with their clinical significances and thus provide opportunities for improving therapies for NDDs in the near future.

**Objective:**

This work aims at a thorough, methodical and critical evaluation of Machine Learning (ML)/Deep Learning (DL) efforts in diagnostics of NDDs, as well as their potential for use in pharmacological, clinical and research settings. An additional objective is to discuss problems and limitations of models, with an eye on furthering research directions.

**Methods:**

The databases of Elsevier, Sage, Wiley, Springer Link, Emerald Insights and Research Gate were searched for useful assessments of NDDs using ML/DL approaches.

**Results:**

Numerous potential diagnostic ML/DL models have been suggested and effectively applied to detect NDDs. Biological, neuroimaging and key clinical characteristics in patients have been used to evaluate prognostic models. Additionally, these models provide options for patient categorization, facilitate earlier detections, enhanced diagnosis and better disease outcome predictions for personalized therapies.

**Conclusion:**

This work points out the rising burden of NDDs on the global elderly while highlighting the significances of ML/DL technological advancements with their limitations for NDDs in diagnostics, identification of effective disease bio-markers and management.

## Introduction

**Figure 1:**
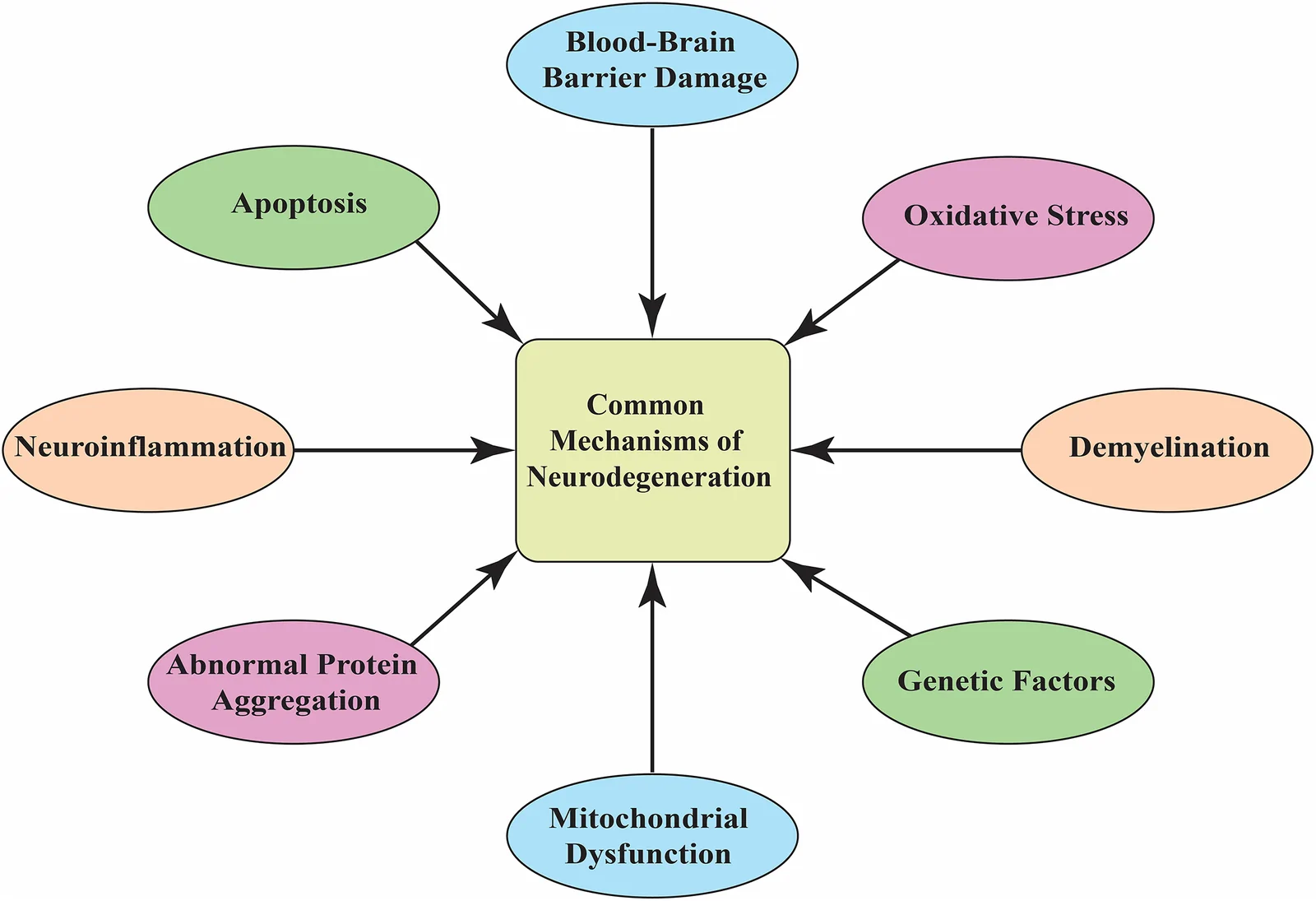
Neurodegeneration mechanisms.

Neurodegenerative diseases (NDDs) are progressive, incurable diseases that cause neurons and nervous system function to gradually decline. Studies indicate that the survival rate for NDDs is low. NDDs afflicted 349.2 million people worldwide (Ding et al., 2022) and are the second most prevalent global disorders. The major forms of NDDs include Huntington’s disease (HD), Parkinson’s disease (PD), Alzheimer’s disease (AD), multiple sclerosis (MS), and amyotrophic lateral sclerosis (ALS). NDDs can be diagnosed using clinical evidence and supportive imaging data from medical modalities (Huang & Zhang, 2023). They mostly affect older persons and are frequently caused by genetic, environmental, or aberrant protein accumulation. They result in memory loss, motor dysfunction (Parkinson’s, ALS), and devastating cognitive decline (Alzheimer’s). The onset of NDDs results in people losing neurons and synaptic connections as they age (Pant, Kapri & Nain, 2022). The progression of the disorders causes the development of clinical symptoms that are closely associated to neuronal death. The hippocampus, a part of the brain involved in declarative episodic memories, loses neurons in AD (Jimenez-Balado & Eich, 2021) while Neuronal myelin sheaths are attacked by immunological responses (microglia) in MS, leading to demyelination and compromised neural signal transmissions. In normal clinical trials of PD, bradykinesia, postural instabilities and tremors appear after 80% of dopaminergic neurons in the substantia nigra are damaged (DeMaagd & Philip, 2015). Many disorders identified by distinctive symptoms that vary according to places of neuronal losses in the brain are shown in Fig. 1 (Gadhave, Sugandhi & Kokare, 2024).

**Figure 2:**
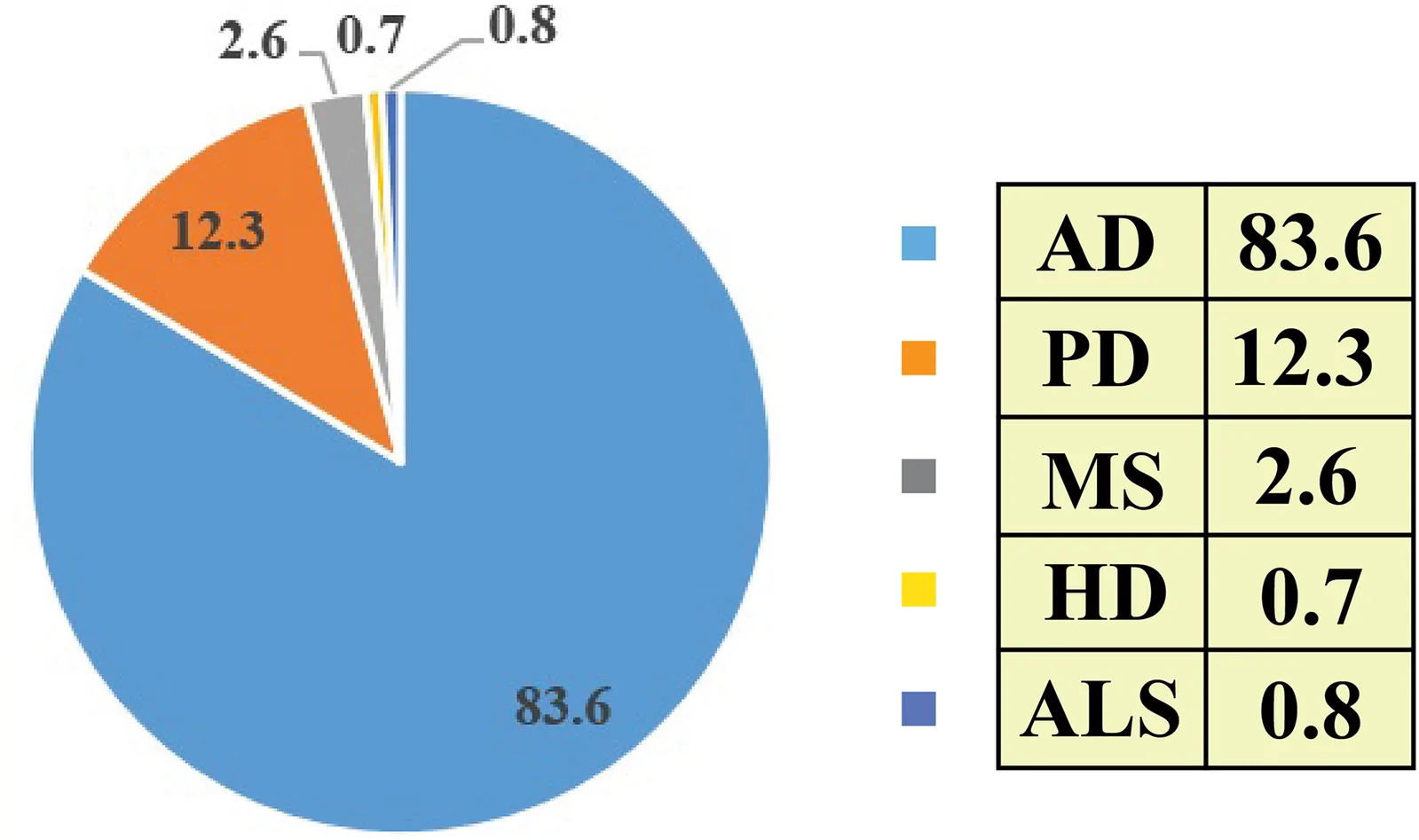
Global prevalence of major NDDs.

HD is a genetic disorder that arises from defective genes copy of parents. Cytosine–Adenine–Guanine (CAG) trinucleotide repeats in the huntingtin (HTT) gene (Saudou & Humbert, 2016). CAG repeats are significantly correlated with age. Family histories of patients are considered in HD diagnostics for identifying distinctive symptoms. Diagnostic genetic tests confirm HTT gene mutations (Schneider & Bird, 2016) in patients. HD cannot be cured, but patients can be managed with supportive care and medications. Prescribed medications can help reduce motor symptoms and enhance overall quality of life in patients. Dopamine is a neurotransmitter that regulates and coordinates specific muscle activities (Gepshtein et al., 2014). The degenerations of neurons producing dopamine in substantia nigra area of the human brain is a common cause of PD where patients who suffer from stiffness, postural instabilities, tremors and bradykinesia (Khatri et al., 2020). PD is mostly diagnosed using clinical evaluations of symptoms and medical history. Neurological tests and imaging techniques may be employed to rule out other conditions. Levodopa which substitutes dopamine is frequently used to treat PD. Dopamine agonists and MAO-B inhibitors are prescribed medications for PD patients (Krishnappa et al., 2022). AD bears the name of Dr. Alois Alzheimer who was the first to describe the disease in 1906. AD is characterized by increase in brain’s aberrant protein aggregates including tau tangles and beta-amyloid plaques resulting in deaths of cells and slow degradations of brain tissues which obstruct normal impulse flows between nerves (Breijyeh & Karaman, 2020). AD symptoms include loss of memory, behavioral changes cognitive declines and restricted functions. The diagnosis of AD is established by medical history reviews, cognitive tests and identification of factors resulting in cognitive declines. In MS, the immune system mistakenly targets myelin covers, protecting nerve fibers (Lamptey et al., 2022). Patients experience fatigue, decreased mobility, sensory abnormalities including tingling or numbness, muscular weaknesses, poor coordinations, visual problems including double or blurred visions and cognitive deficits that affect memory (Coles, 2015). Genetic or environmental elements related with smoking, low vitamin D levels and other illnesses can cause MS in patients (Sugandhi et al., 2024). The slow degenerations of brain and spinal cord nerve cells, referred to as Lou Gehrig’s disease, is the hallmark of ALS. Human brain fails to regulate muscle movements when motor neurons degrade. The disorder is diagnosed using defined clinical symptoms and methodical eliminations of other possible causes. AD is the 6^th^ most common cause of global mortalities. Where above 60% of these cases occur in low/middle-income countries (Naheed et al., 2023). PD affects 8.5 million with 329,000 fatalities with an increase of 81% since 2000 (Ou et al., 2021). MS accounts to 1.8 million with 22,439 deaths and 59,345 new cases (Wallin et al., 2019). MS related mortalities and ALS incidences (1.9–6 cases for every 100,000) have been steadily increasing ALS’s mortalities based on age were 24,328 deaths or 1.70% (Larson et al., 2018). Global HD prevalences were 2.71/100,000 individuals with an annual incidence rate of 0.38/100,000. HD deaths attributed to 2.27/million annually. Although there are several authorized treatments for NDDs, they have failed to control the course of NDDs (Niazi, 2023). Most of them merely aid in treatment of symptoms linked to the disorder (Gadhave et al., 2023). Figure 2 shows the prevalence of major NDDs (GBD 2019 Mental Disorders Collaborators, 2022).

Genetic counseling is recommended for understanding potential consequences (McGarry et al., 2020). Thus, raising awareness about NDDs is essential for supporting affected individuals and expanding researches in this domain. Though numerous research articles on NDDs utilizing ML/DL classifiers and predictive modeling for NDDs have been published, their shortcomings are rarely examined. Hence, this work attempts to highlight these shortcomings suggest future paths for diagnostics of NDDs using ML/DL approaches.

## Methods

The rapidly emerging fields of ML/DL aim to design and use computer programs that can learn on their own. Their learning often starts with sets of instructions and training on data sets. ML/DL applications in healthcare have shown promise in risk classifications, diagnosis and prognosis (Kourou et al., 2015).

**Objectives:** This work evaluates usefulness of ML/DL techniques in diagnostics of NDDs, disease connected factors and areas of improvements for ML/DL techniques.

**Study design and search strategies:** Studies were thoroughly searched for NDDs and ML/DL works that had previously been shown to be helpful for NDDs in Research Gate, Elsevier, Sage, Wiley, Springer Link and Emerald Insights. The right databases were examined to prevent duplications. The retrieval of publications were aided by previously recognized references. The searches were based on medical subject headings (MESH) terms of NDDs and their associated ML/DL applications including debates. This study estimates the significance of AIs, specifically ML/DL implementations in diagnosing NDDs while adhering to Preferred Reporting Items for Systematic and Meta-Analysis (PRISMA) protocols.

**Eligibility Criteria:**

**Criteria for Inclusions:** The following conditions were considered for including publications in this work

(1) **Populations:** Studies analysing NDDs based on data/datasets relevant to humans.
(2) **Study Designs:** All observational designs including cross-sectional, datasets and case-control studies were considered for this work.
(3) **Configurations:** Full-text articles available to the general public.
(4) **Studies:** All published and unpublished works, such as journal articles, master’s theses and dissertations between 2015 and 2025 were taken into account for analytics.
(5) **Languages:** All articles in English only were considered for this work.

**Criteria for Exclusions:** Non-empirical works including review articles, comments, research publications and theoretical frameworks without primary data that were unrelated to usages of ML/DL techniques for NDDs.

**Figure 3:**
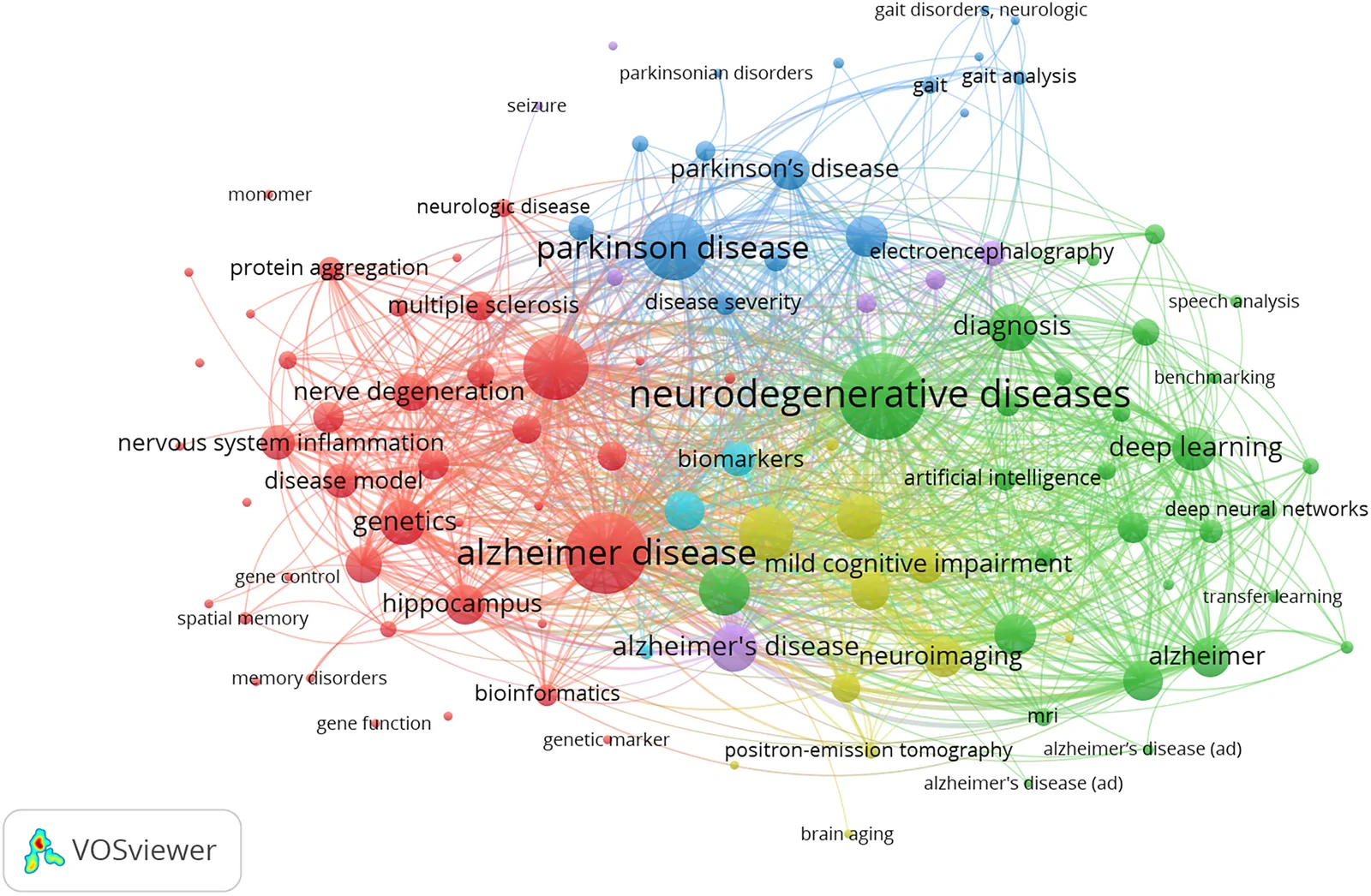
VOS viewer based bibliometric analysis visualization.

**Quantitative assessments with data collections:** Quantitative approaches use statistical and mathematical techniques to analyze scientific literature to find research trends, patterns and impacts where this work used bibliometric analysis (Refer Fig. 3) to understand the “who,” “what,” and “where” along with its evolution. The analyses find research gaps, assess effectiveness of studies, journals and institutions, and map field structures. They also examine features of documents such as articles, reviews, and conference proceedings. Titles and abstracts were used to determine each article’s eligibility for the review based on predetermined standards. The quality of the research was evaluated using criteria that were modified to account for cross-sectional studies and reporting prevalence statistics. Data collection, cleaning, and analysis protocols were followed. Microsoft Excel and a specialized program called VOS Viewer were used to process the data and create tables and infographics. Any disagreement that emerged throughout the review process was resolved by talking with other people.

**Data extraction:** Information on titles, authors, years of publications and designs were extracted using author’s self developed program. Articles that satisfied the predefined criteria provided the data for the final analysis. Disparities during extraction of studies were overcome by the reviewer to ensure consistency. Inconsistencies were handled by speaking with other authors and repeating the aforesaid procedures.

**Methodical Assessments of Qualitative Studies:** The quality of this work was ensured by assessments of research involving patients with NDDs and using pre-established and modified checklists. If an item met the methodological norms, it earned a positive score. Items that did not match the methodological standards received a no score, while those that did not meet the criteria but were unable to fully characterize the relevant parameter earned an unclear score. The publications that matched the criteria for a systematic review and meta-analysis were screened using the aforementioned keywords. The limitations of this meta-analysis and systematic reviews were on answers “no” or “neither yes nor no,” which cannot be read. A score of four or higher on the quality assessment checklist was considered suitable for measuring bias risk in cross-sectional research. Quality in this work was evaluated on answering the below stated questions.

- Q1: Were the study materials sampled properly?
- Q2: Were included articles and environments detailed?
- Q3: Did analytics cover the identified samples adequately?
- Q4: Were legitimate procedures used to identify conditions?
- Q5: Were criterions measured with consistency?

**Outcome of interest:** The main outcome of this meta-analysis and systematic review was the global prevalence of NDDs while risk factors of NDDs and studies related to NDDs with usages of ML/DL techniques was the secondary outcome.

**Heterogeneity and Publication bias:** Heterogenous test results amongst studies on NDDs were equally classified.

**Data processing and analysis:** Despite their better accuracy over classic ML models, DL models’ generalizability was hampered by lack of clinical validations and diverse datasets. Hence, trends of dataset reliance, multimodal data integrations and clinical validations were examined in models detecting NDDs.

## Results

**Figure 4:**
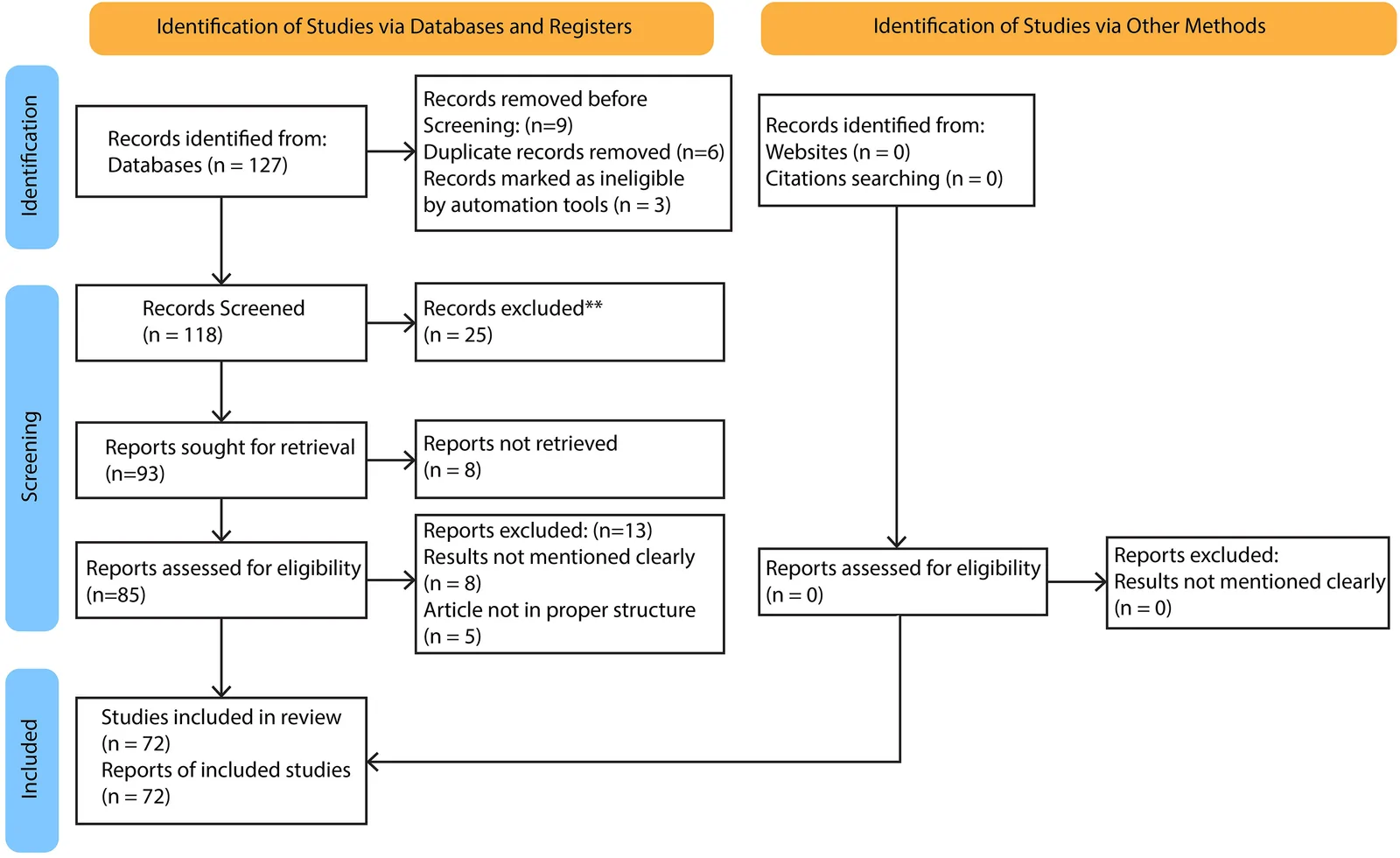
PRISMA diagram of selected and included ML/DL studies for NDDs in systematic review and meta-analyses.

**Studies identified:** Out of the 127 records identified from the database search, nine were removed before screening due to duplicate records (*n* = 6) and automatic eligibility marking (*n* = 3). Of the 118 records screened, 25 were excluded based on title and abstract, and out of 93 reports requested eight were not retrievable leaving 85 reports for full-text review. Of the eligibility reports assessed, 13 were excluded because the results were not mentioned (*n* = 8) or the article did not have a clear structure (*n* = 5). As a result, 72 studies were identified for the review. Additional records to be assessed were not found from other strategies, such as, searching the web or citation searching, therefore additional reports were not included or excluded from these strategies. The use of ML/DL in diagnosing NDD was recommended by the criteria (Fig. 4).

## ML/DL in AD diagnostics

**Figure 5:**
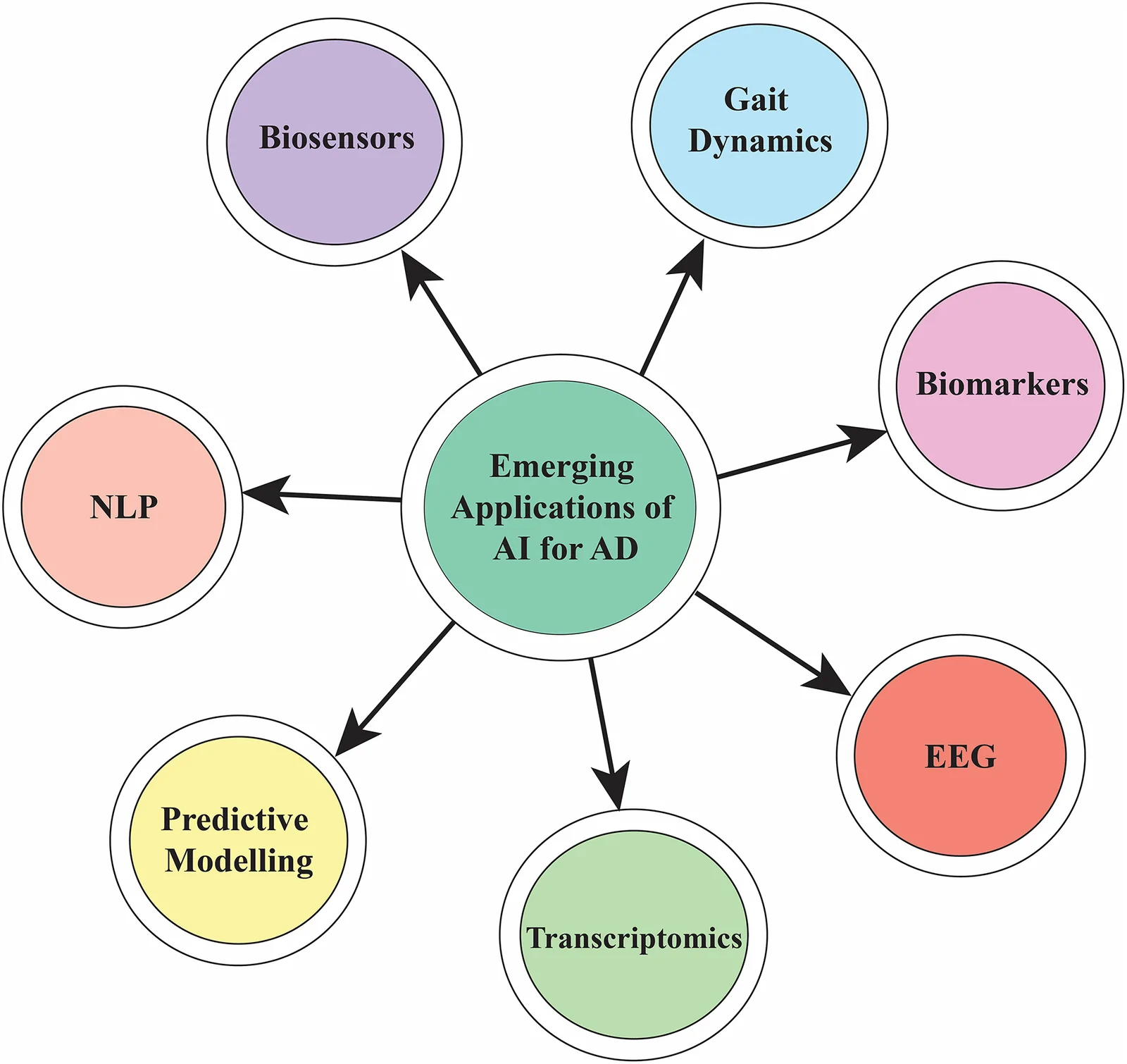
Emerging applications of AI/ML for AD.

Early identification of AD becomes difficult due to significant inter-individual heterogeneity, sluggish disease development and overlapping symptoms with other NDDs (Basha et al., 2025). ML/DL techniques have become effective tools for overcoming these diagnostic constraints. They can analyse high-dimensional or multimodal biological data including molecular biosignatures, clinical narratives, neuroimages, gait dynamics and genomes (see Fig. 5). ML/DL models uncover hidden patterns in support of clinical disease indicators and assist in early detections, risk stratifications and personalized monitoring of AD patients.

### Transcriptomics and immunity biomarkers

Transcriptomic profiling of AD is linked to extensive changes in gene expression that include oxidative stress, immunological dysregulations, and controlled cell death pathways, where gene expression data’s high dimensionality presents significant analytical difficulties in transcriptomic investigations. Current studies combine predictive performances with biological interpretability for biomarker identifications using AI-based ML/DL techniques to overcome the restrictions of traditional statistical models. They select features, reduce dimensionalities and classify AD to find immune-related biomarkers in diagnostics. Blood expression datasets (GSE63060, GSE63061, and GSE97760) were analyzed in the immune-focused transcriptome investigation of He et al. (2022). Differential expression analyses were combined with weighted gene co-expression network analysis (WGCNA) identified gene modules linked to the disease. Nonlinear gene connections in high-dimensional transcriptome data were selected using random forest (RF). The selections were made based on intrinsic feature importance ratings, and seven hub genes were discovered and analyzed for biological relevance. The chosen genes were then utilized to create an artificial neural network (ANNs) that replicated complex, nonlinear expression patterns. The schema’s assessments resulted in area under the curve (AUC) values larger than 0.80. Cross-validations assessed performance variability and confirmed the diagnostic model’s durability. ANNs were selected due to their ability to combine several linearly inseparable immune-related signals. The work in Deng et al. (2022) used WGCNA, differential expression analyses and gene set enrichment analysis (GSEA) to identify ferroptosis associated genes of AD from brain transcriptome datasets (GSE33000, GSE5281 and GSE48350). For feature selections, many ML techniques such as support vector machines (SVM), RF, XGBoost, least absolute shrinkage and selection operator (LASSO) were employed. Their L1 regularizations promoted penalty for sparsity, minimizing data overfits and producing interpretable compact gene sets, five genes (NFKBIA, RAF1, FOXO1, MOV10L1 and IQGAP1) were found by combining the intersection of properties found using various ML techniques into a multivariable linear regression (LR) model. AUC values for the diagnostic model were 0.80 in external validation datasets, 0.94 in training sets and 0.96 in test sets. The work’s multistep analytical procedure demonstrated better performances of LR selected features when compared with differential expressions or co-expressional metrics only. The study in Liu et al. (2022) employed transcriptomic ML frameworks for identifying major depression disorders on GSE19738 and GSE98793 blood datasets. Following differential expression analysis, a network of protein-protein interactions was created, with hub genes identified. RF correlated predictors and ranked gene importance during feature selection. ANNs are categorized using networks made up of specified characteristics. The model scores of AUCs were 0.757 and 0.685 in the training and test sets. This work despite poor accuracy demonstrated the wider application of an AI-based transcriptome diagnostics, highlighting issues in modeling various mental disorders using peripheral blood data. Three brain transcriptome datasets were combined in Alamro et al. (2023) to identify AD biomarkers using a thorough feature selection and model comparison approach. The study used regularization based LASSO and Ridge as feature selection techniques, after initially identifying hub genes based on ranking six protein–protein interaction networks. The selected approaches handled multi-collinearities and minimized model complexities when compared with unregularized alternatives. LASSO eliminated variables while Ridge was instrumental in reducing correlated coefficients. Genes selected by LASSO and Ridge, obtained an AUC of 0.97, showing better performance when compared with feature sets. Subsequently, trained classifiers evaluated the discriminative abilities of the gene subsets. Statistically driven features performed better in terms of classification accuracy when compared to network topologies relying on gene rankings. They produced compact, stable, and highly discriminative gene sets. Thus, ML/DL techniques have been of assistance in discovering transcriptome markers associated with iron dysregulation and ferroptosis in AD.

### NLP and predictive modeling

The use of structured and unstructured clinical data for predictive modeling of AD diagnosis has been made possible by the growing availability of electronic health records (EHRs) where ML and natural language processing (NLP) models evaluate AD. These methods complement traditional clinical evaluations by focusing on early risk classifications. The work in Mao et al. (2023) suggested AD-Bidirectional Encoder Representations from Transformers (AD-BERT), a transformer-based NLP approach for predictions of AD progressions using unstructured clinical notes from EHRs. On initialization with Bio + Clinical BERT, the model was pretrained on clinical narratives related to AD for extracting illness specific language patterns. Global max pooling was used into patient-level representations and assess and encode clinical notes acquired at or prior to the initial diagnosis of mild cognitive impairments (MCI). A fully connected classifier was then used where AD-BERT achieved an area under the ROC curve (AUC) of 0.84 on Northwestern Medicine (NMEDW) dataset and 0.88 on external Weill Cornell Medicine dataset, outperforming several baseline NLP and ML models. The outcomes supported the use of transformer-based models for NLP, particularly in cases where narrative evidence pointed to long-term alterations in cognition and function. Similarly, the work in Formica et al. (2023) employed a supervised ML model that forecasted cognitive declines in AD individuals based only on organized clinical, neuropsychological and demographic data. The authors used a limitted test set (*n* = 60) with a larger training dataset (*n* = 4,848) to build their classifications on RoboMate platform. The model’s score in diagnosis accuracy was 86% and in DeLong’s test, Mini-Mental State Examination (MMSE) values greatly improved discrimination compared to using only clinical criteria.

### Gait dynamics and spatiotemporal biomarkers

Gait analysis can be used in non-invasive diagnostics for AD, as motor control impairments frequently appear alongside visible cognitive deteriorations. Technological advances in wearables, sensors, cameras, and ground reaction force (GRF) devices have made extractions of spatiotemporal gait characteristics like stride lengths, cadences, stances, and swing timings possible. Gait characteristics for different circumstances, such as walking, can also be captured using these devices. These characteristics can deliver unbiased motor function assessments using ML/DL techniques. DL models, convolutional neural networks (CNNs), and recurrent neural networks (RNNs) evaluated gait data obtained from wearables and motion capture devices in Sarkar (2025). The designs simulated temporal dynamics (RNNs) and spatial dependencies (CNNs) observed in gait sequences, where their results showed better performances when compared to conventional, non-AI diagnostic techniques. Though the work attained a detection accuracy of over 90%, only overall accuracy assessments were provided, and confidence ranges and uncertainty estimates for model performances were omitted. The study in Seifallahi, Galvin & Ghoraani (2024) used skeletal joint data obtained from Kinect v.2 depth camera to analyze gait patterns when patients walked ovally and straight. Several ML approaches were investigated, including RF, logistic regression (LR) and SVM. The capacity of RF classifiers to handle nonlinear interactions between gait data, as well as their robustness to feature correlations, influenced their selection. The study found that more complicated gait circumstances, such as oval-path walks, showed novel discriminative features for MCI identification. For MCI classifications, the model obtained 85.5% accuracy and an F1-score of 83.9%. These results confirmed task-specific gait paradigms, although they were unclear concerning performance variability and confidence intervals. To assess raw GRF signals and manually produced spatiotemporal attributes at the same time, NDDNet of Faisal et al. (2023) was a modified DL architecture that incorporated temporal CNNs and bidirectional long short-term memory (LSTM) layers. LSTM layers simulated long range temporal dependencies in gait cycles while convolutional extracted local features. NDDNet’s mean classification accuracy for categorizing PD, HD and ALS, was 96.75%, which demonstrated the usage of gait-based artificial intelligence (AI) models for multi-disease screening. However, confidence intervals and other performance uncertainty indicators were not provided. Collectively, these studies show that clinically significant motor changes linked to cognitive impairment and neurodegenerative illness may be captured by AI-assisted gait analysis.

### Structural imaging with DL

Structural magnetic resonance imaging (MRI) capacity to identify patterns of cortical and subcortical atrophy specific to an area, makes it a fundamental technique for describing NDDs. ML/DL-based extractions of quantitative structural information from MRI can automate extractions of features, handle high-dimensional data, and generalize across datasets. Twenty nine patients with tremor-dominant PD (tdPD) and 61 patients with essential tremors (ET) had their structural T1-weighted MRI data examined using an AI-based brain volumetry approach in Purrer et al. (2023). The DL was trained on U-Net-segmented normative database features, where cortical and subcortical region volumes were modified based on internal reference controls for age and sex, resulting in standardized deviation maps. Their schema identified occipital lobes, putamen, pallidum, mesencephalon, and hippocampal regions aberrant in both ET and tdPD individuals. The study found Thalamic and caudate nuclei smaller in ET patients. The work demonstrated DL based volumetry estimates for regional atrophy patterns important to the diagnostics of NDDs. Similarly, Noella & Priyadarshini (2023) employed generative adversarial network (GAN) in conjunction with DCNN (GAN-DCNN) to categorize PD, AD and frontotemporal dementia (FTD) from FDG-PET brain images. The dataset comprised of 160 images from Non-fluent Variant Primary Progressive Aphasia Dementia (NIFD) groups and 350 scans from Parkinson’s Progression Markers Initiative (PPMI), AD Neuroimaging Initiative (ADNI), and normal controls. GAN components created synthetic positron emission tomography (PET) images for reducing class imbalances. Deep CNNs (DCNNs) classifications of instances attained 97.7% in sensitivity and 0.97 in specificity, after learning. Their 95% confidence interval for accuracy assumed binomial distributions and was based on reported sample sizes of 95.9–99.0%. The study emphasized usage of GANs to address data imbalances, a common restriction in datasets of NDDs. However, interpretability analyses were excluded and external validation datasets were missing in the study. MUQUBIA (De Francesco et al., 2023) was an explainable ML system based on SVM that differentiated between AD, FTD and dementia with Lewy bodies (DLB). Five hundred and six participants from five databases—ADNI, FTLDNI, NACC, PDBP and Newcastle—were included in the study. The work utilised structural MRI derived characteristics and clinical data to generate cognitive normal controls where FreeSurfer, LPA and TRACULA pipelines were used in analyses. MRI data were used to compute diffusion metrics, white-matter lesion burdens, cortical thicknesses, and subcortical volumes. Their optimal input sets with ages, genders, Clinical Dementia Rating (CDR) and 19 imaging parameters were identified in feature selections. MUQUBIA obtained an accuracy of 88% and an AUC of 0.98 in the test dataset. Unsupervised ML technique of Qin et al. (2023) detected subcortical ischemic vascular diseases (SIVD) and subcortical vascular cognitive impairments (SVCI) patients. Forty five SVCI and thirty two SIVD instances were externally validated. Their internal dataset had eighty three SVCI and fifty three SIVD healthy instances. Their features without labels encompassed diffusion tensor images (DTI), T1-weighted MRI, and resting-state functional magnetic resonance imaging (fMRI) instances. The schema attained values of 86.03%, 79.52%, 96.23% in internal and 80.52%, 71.11%, and 93.75% in external datasets respectively for accuracy, sensitivity and specificity. Generative pre-trained transformer 4 vision (GPT-4 V) was investigated in Ono, Dickson & Koga (2024) and contrasted with YOLO v8 CNN model in order to diagnose tau pathology in histology images. The model using a dataset of 1,520 images with AD, PSP, and corticobasal degenerations, obtained 90% accuracy after 20 shots of learning and 40% accuracy in zero shots. The authors cautioned that task-specific CNNs are still required for neuropathological diagnostics and textual contexts had significant influences on GPT-4 V performances.

### AI-powered biosensor technologies

Complex, high-dimensional spectroscopic/electrical datasets produced by advanced biosensors are not completely interpretable by traditional univariate or rule-based statistical techniques, where AI integrations with biosensors for diagnostics of NDDs have emerged as responses. ML/DL algorithms can extract quantitative, therapeutical and meaningful data from biosensors for different co-existing biomolecular states or biomarkers. PD related α-synuclein (aSyn) species were identified from immunoassay coupled surface enhanced infrared absorption (ImmunoSEIRA) biosensors in Kavungal et al. (2023). Time resolved infrared absorbance spectra in amide I/II regions of protein were assessed using microfluidics and a nanoplasmonic gold metasurfaces. ASyn can be selectively enriched from solutions *via* antibody-based captures, producing spectrotemporal datasets and displaying fibrillar, oligomeric and monomeric conformations. deep neural network (DNN) was used as a multilayer perceptron (MLP) and trained on raw, unprocessed spectrotemporal data derived from tightly controlled oligomer and fibril pairings. The network used four hidden layers, 133 spectral inputs and five-fold cross-validations to improve performances. AI computations of fibril concentrations from defined combinations with predictive accuracy of 94.66%, suggest that AI can manage aggregations of infrared spectral patterns, which are otherwise challenging tasks for traditional methods. The issue of clinical multivariate biomarker integrations were handled in Wang et al. (2024). The work’s femtomolar detections of AD’s plasma biomarkers including Aβ40, Aβ42, phosphorylated tau (P-tau181 and P-tau217) and neurofilament light chain (NfL), using an ultrasensitive graphene field-effect transistor (gFET) biosensors, demonstrated extensions of AI assisted biosensing. Measurements were taken from multicenter clinical datasets. Even though every biomarker showed patterns associated with the disease, single-biomarker studies only exhibited limited discriminating capability. Due to this limitation, ML models were used to concurrently integrate several biomarkers with clinical attributes including age, sex and education. The study concluded that their “composite-info” model based on RF was the most successful after their thorough evaluations of LR, MLP and RF models. Studies related to AD detections are summarized in Table 1.

**Table 1:** Summary of studies on AD.

Data/modality/studies	ML/DL techniques	Findings	Clinical significance	Boundaries/limitations
Peripheral blood transcriptomics (GSE63060, GSE63061, GSE97760 GSE19738, GSE98793) (He et al., 2022; Liu et al., 2022)	RF, ANN	ANN model found seven hub genes (immune related) and validated across multiple datasets. Diagnostics of immune-related DEGs for identifying depressive illness	Demonstrated the use of transcriptome ML to identify mental disorders, promoting non-invasive blood-based immunological biomarkers for AD identifications	Used retrospective designs, mechanistic validations were insufficient, and confidence intervals were absent. Generalizability was limited with poor predictive performances
Brain transcriptomics (GSE33000, GSE5281, GSE48350 GEO datasets (Deng et al., 2022; Alamro et al., 2023)	LASSO, RF, SVM, XGBoost, LR, Ridge Regression	RAF1, NFKBIA, MOV10L1, IQGAP1 and FOXO1 (ferroptosis-genes) developed the diagnostic model. Overlapping genes from Ridge and LASSO discriminated AD from controls.	Identified ferroptosis molecular markers (tiny gene signatures with strong discriminative abilities) for diagnosing AD.	Limited clinical translations of brain tissues; used cross-sectional datasets estimates for uncertainties were absent Required more clinical validations. Confidence ranges were absent.
EHR NMEDW and WCM datasets (Mao et al., 2023)	AD-BERT, Clinical BERT	AD-BERT outperformed other baseline NLP and ML models in predicting AD on both datasets	Supported usages of EHRs for NLP based predictions and demonstrated utilization of clinical notes for early risk stratifications in MCI patients.	No biomarker data, documentation inconsistencies leading to performance differences across systems, reliance only on ICD codes for describing outcomes, AUC based implicit reports for confidence ranges.
MMSE, comorbidities, functional scales (Formica et al., 2023)	ML on RoboMate platform	Clinical characteristics with MMSE differentiated MCI from AD when compared clinical characteristics along.	Provided clinical screening and second opinions with risk categorizations and without biomarkers or complex imaging.	Very little data *n* = 60 was used, absence of longitudinal validations and reliance on MMSE of neuropsychological elements.
Wearable sensors and motion capture–derived spatiotemporal gait features (Sarkar, 2025)	CNN, RNN	By utilizing gait dynamics to differentiate between healthy individuals and early AD, DL models surpassed conventional diagnostic techniques.	Encourages gait-based, non-invasive early AD screening.	Confidence intervals are not disclosed, and the dataset’s size and variability are not made publicly available.
Kinect v.2 skeletal joint data (Seifallahi, Galvin & Ghoraani, 2024)	RF, SVM, LR	Oval walks showed better discriminative gait patterns detecting MCI when compared to straight walks.	Demonstrated the effectiveness of task-specific gait tests in early MCI screening.	Modest sample sizes; lack of uncertainty or external validation estimates.
GRF signals, spatiotemporal features (Erdaş, Sümer & Kibaroğlu, 2023)	NDDNet	A single DL architecture was used to accurately classify a number of NDDs based on gait.	Demonstrated possibilities of multi-disease gait-based screening.	Absence of uncertainty measures; AD detection was not the primary goal.
Structural MRI (T1-weighted), clinical variables, DTI, resting-state fMRI (Purrer et al., 2023; De Francesco et al., 2023; Qin et al., 2023)	U-Net, SVM, SHAP, Unsupervised ML	In addition to thalamic and caudate atrophy, ET patients also showed overlapping volumetric abnormalities in the hippocampus, putamen, pallidum, mesencephalon and occipital lobes. Identified morphological markers specific to hippocampal shrinkage in AD and differentiated between individuals with AD, FTD, DLB and cognitively normal. Patients with SIVD who were at risk of developing SVCI were identified using multimodal MRI features.	Demonstrated structural changes associated of diseases using AI-driven standardized MRI volumetry. Combined diagnostic performances with interpretability to increase physician confidences and acceptability. Provided an easy MRI-based technique for predicting vascular cognitive impairments.	Absence of diagnostic classifications, AD was not included, absence of accuracy metrics or confidence ranges in. MRI preprocessing procedures; Considered clinical aspects and images with limited mechanistic explanations and long-term validations.
FDG-PET (Noella & Priyadarshini, 2023)	GAN, DCNN	AD, PD and FTD, including early-stage AD, were effectively classified by PET imaging.	Demonstrated exceptional diagnostic accuracy when using single imaging modalities in categorizing several dementias.	Lack of external validations, limited interpretability and possible bias from artificial data.
Neuropathological histology images (Ono, Dickson & Koga, 2024)	GPT-4Vision, YOLOv8 CNN	GPT-4 V performances improved with limited shot learning, equalling accuracy of CNNs.	Showed usages of multimodal LLMs in neuropathology with minimal labels.	Highly context-sensitive non-MRI-based methods making them inappropriate for diagnostics.
Time-resolved infrared absorbance spectra bands (amide I/II) from immunoSEIRA plasmonic biosensors (Kavungal et al., 2023)	DNN, MLP	The proportion of fibrillar α-synuclein in regulated oligomer–fibril mixes was accurately predicted by AI-enabled regressions, outperforming conventional spectral analysis for mixed data.	Allowed quantitative assessments of structural protein aggregations overcoming limitations of existing structural biomarker based approaches.	No external validations or uncertainty measurements of *in vitro* protein combinations and no testing of clinical datasets.
Electrical responses of plasma Aβ40, Aβ42, P-tau181, P-tau217 and NfL (Wang et al., 2024)	RF,LR, MLP	The combined biomarkers enhanced differentiations between AD, MCI and cognitively normal patients when compared to singular biomarkers.	Demonstrated viability of blood-based AD screening and staging with AI based biosensor data.	Moderately sized dataset; results applied only to the studied population and no external validation datasets were used.

## PD diagnosis/monitoring using ML/DL

**Figure 6:**
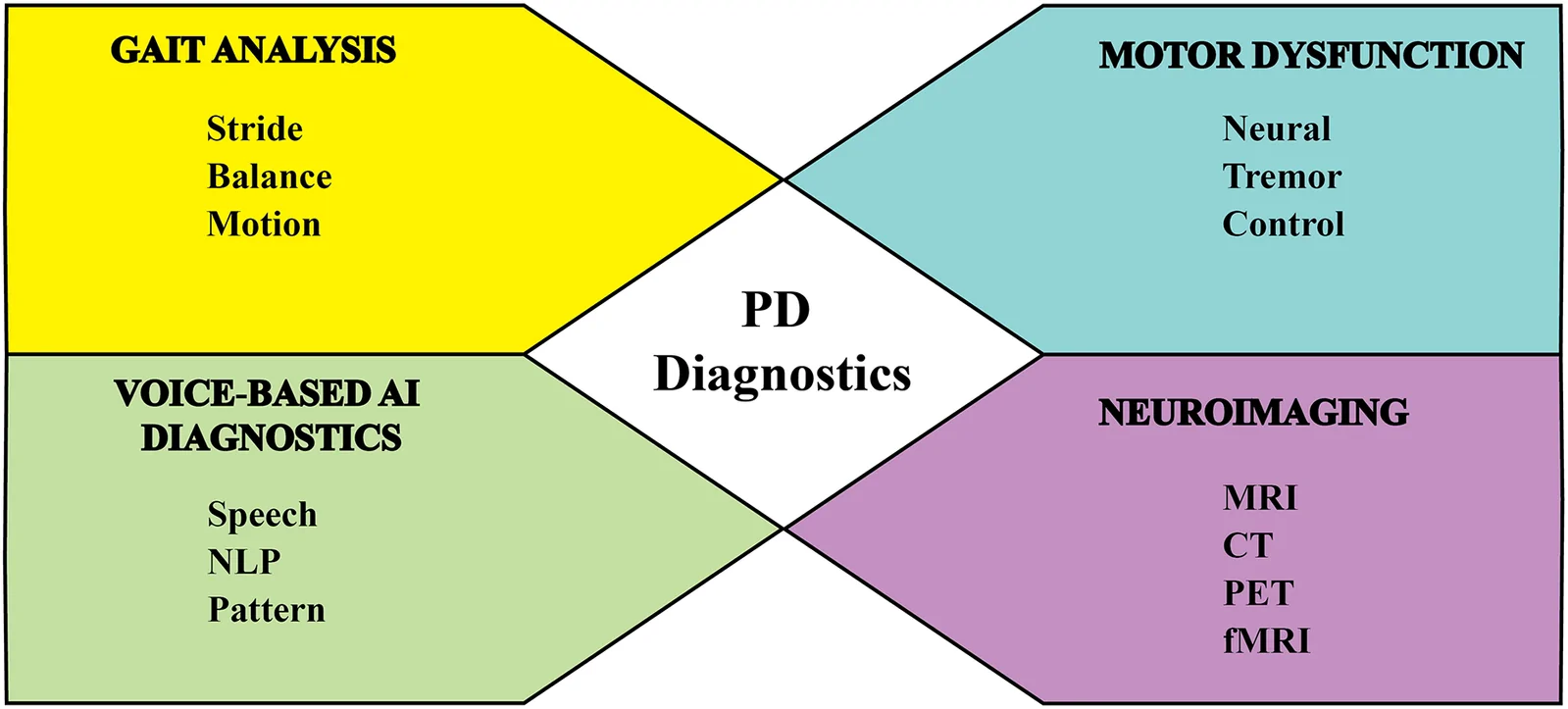
PD diagnostics using multimodal data.

AI utilizing data-driven, objective methods that complement traditional clinical evaluations has evolved into a revolutionary tool for diagnosing PD. The limitations of symptom-based diagnosis can be overcome by using AI-driven models to uncover subtle, disease-specific patterns in complex biological and behavioral data, given the variability of PD symptoms. Voice-based diagnostics, motor and gait analysis and neuroimaging-assisted detections for PD diagnosis are the three main topics covered in this section. The investigations in this area are summarized in Fig. 6 shows multimodal data-based PD diagnoses.

### Voice-based AI diagnostics for PD

PD’s motor symptoms appear when 60–80% of dopamine synthesis gets exhausted, causing death of dopaminergic neurons. Physical and psychological evaluations with Neurological tests are a major part of PD’s clinical diagnosis. Recently, voice-based analyses have become viable non-invasive methods its early identifications, since speech and phonatory problems are common in people with PD. ML based voice diagnostics seek to comprehensively model PD stage related speech patterns, complementing traditional clinical evaluations. ML pipeline of Erdaş, Sümer & Kibaroğlu (2023) addressed dataset imbalances using synthetic minority over-sampling technique (SMOTE) and used recursive feature elimination (RFE) to assess feature relevances. Many classifiers, including SVM, K-nearest neighbors (KNN), decision trees (DT), RF and MLP assessed feature sets. Principal component analysis (PCA) and t-distributed stochastic neighbor (t-SNE) reduced data’s dimensionalities. MLP and PCA in combinations achieved an accuracy of 98%, while RF and t-SNE combination’s scores for accuracy were 97%. The work in Alalayah et al. (2023) distinguished healthy controls from untreated PD and mid-advanced PD patients receiving L-DOPA therapies using acoustic biomarkers while Costantini et al. (2023) employed 1/2D CNNs in conjunction with pretrained models of Wav2Vec 2.0 (voices) and BERT/BETO (languages) for distinctions. Speech and language impairments of patients were modeled first and subsequently, early, joint and late fusions were combined. Models for speech distinctions showed an accuracy of 88% consistently, outperforming language based and multi-modal representations. Their results implied that temporal and representational scale matches were a necessity in speech and language biomarker combinations and that increased modality coverages do not always imply enhanced diagnostic performances. The work in Escobar-Grisales, Ríos-Urrego & Orozco-Arroyave (2023) developed a strong multi-class ML system capable of distinguishing patients with PD and spasmodic dysphonia from healthy controls. The study evaluated 6,373 acoustic data elements from one hundred and eleven healthy persons, fifty one PD patients and sixty dysphonic patients. The study’s conclusions were based on standardized Italian phrases and sustained phonation of vowels. Moreover, the study highlighted the need for multi-class evaluations in voice-based AI diagnostics by including differential diagnosis into their modeling frameworks. The study in Cesarini et al. (2023) investigated ML techniques for voice analysis in PD diagnostics. The work evaluated eighty one speeches using a hybrid model consisting of CNNs, RNNs, multiple kernel learning (MKL) and MLP architectures. The model’s inputs were acoustic features including jitter, shimmer and Mel-Frequency Cepstral Coefficients where the suggested model obtained 91.11% accuracy with corresponding values of 92.50% for recall, 89.84% for precision and 91.13% for F1-scores. Their AUC of 0.912 with SHapley Additive Explanations (SHAP) values enhanced model’s transparency by emphasizing on features with greatest categorization impacts. The study in Shen, Mortezaagha & Rahgozar (2025) suggested an integrated diagnostics using Quantized Contempo Neural Networks (QCNN) for classifications and Adaptive Intelligent Polar Bear (AIPB) for optimizations. Determinate Haar Wavelet (DHW) transformations preprocessed voice data to minimize noise and improve the signal’s qualities. Statistical Time-Frequency Renyi (STFR) model selected features, which were optimized by AIPB before QCNN classifications. This study demonstrated trade-offs between computational complexities and algorithmic sophistications in speech-based PD detections while suggesting alternative design strategies. The work in Pragadeeswaran & Kannimuthu (2024) tested multiple classifiers including KNN, SVM, RF and eXtreme Gradient Boosting (XGBoost), using similar ensemble combinations on one hundred and ninety five instances of a PD voice dataset. The work in Mahesh et al. (2024) examined ML based feature selections for detecting PD in 197 voice samples with twenty three acoustic characteristics. The DT classifier with an accuracy of 94.87% and receiver operating characteristic (ROC) value of 98.7%, demonstrated outstanding resilience in smaller and complete feature sets.

### Sensor based AI approaches for PD

Sensors are not just pattern recognizers; they can also be used as analytical instruments. The work in Yadav, Singh & Pal (2023) used a wearable triboelectric nanogenerator (W-TENG) recycled nylon-66 sensors with ML algorithms to categorize PD movement patterns. The obtained movement-derived voltage signals were explored using K-Means clustering (KMC), demonstrating a scientific link between brain diseases and peripheral motor sensing. The study in Babu et al. (2023) examined recorded acoustic characteristics of PD patients using SVM, which differentiated PD patients from healthy controls. The schema’s F1-score was 0.87. Handwriting and neuroimaging evidences of motor impairments are functionally related, making them sensitive markers for basal ganglia dysfunctions. The work in Krishnappa & Shashidhar (2023) utilizing spiral and wave drawings suggested the use of explainable artificial intelligence (EXAI) models for detecting PD early. Theit combination of VGG19 and Inception based networks, learnt from dataset of drawings to classify healthy controls and PD patients with 98.45% accuracy. The model addressed “black-box” issues of DL techniques in clinical settings by integrating Local Interpretable Model-agnostic Explanations (LIME) and demonstrated identification of decisive elements from drawings. The work in Saravanan et al. (2023) methodically addressed several analytical issues for PD detections using multiclass ML/DL models on speech signal. The schema used SMOTE for balancing classes, feature selections and Randomized SearchCV for hyperparameter tuning. Classification of instances were based on Feed-forward neural networks (FFNN) and kernel-based SVMs where FFNN achieved 99% accuracy. These studies demonstrate the use of important biomarkers for PD diagnostics, applicability of movement sensors and motor evaluations based on handwriting.

### AI-driven neuroimaging methods in PD diagnostics

ML/DL techniques based on neuroimages for PD diagnostics have shown promise as supplements to clinical examinations. They have resolved uncertainties found in the early stages of PD, differentiating them from atypical Parkinsonian illnesses. The work (Srinivasan et al., 2024) suggested an AI framework using neuroimages and clinical assessments from the PPMI database. The study used 7,500 MRI images obtained between 2022 and 2023 from people aged between 40 and 85. Transfer learning techniques based on pretrained CNNs developed on ImageNet handled structural MRI data, where Transfer learning helped alleviate lack of disease-specific labeled neuroimaging data. DenseNet169 with a classification accuracy of 85.08% for PD outperformed other evaluated architectures. The work in Islam et al. (2024) described a well-planned, prospective, multicenter study that employed diffusion MRI in combination with linear SVM classifiers to automate Imaging differentiations for PD diagnostics. The study used data of two hundred and forty nine patient’s data with PD, multiple system atrophy, and progressive supranuclear palsy (PSP) obtained from twenty one specialized movement disorder clinics. Their predictions matched postmortem diagnostics in 93.9% of cases, demonstrating the biological validity of the schema. The works demonstrate different but compatible analytical approaches in neuroimaging AI for PD. Table 2 is a summary of ML/DL studies for PD.

**Table 2:** ML/DL studies for PD.

Data/modality/study	ML/DL methods	Findings	Clinical importance	Limitations
Acoustic features, Voice, language recordings Speech (Yadav, Singh & Pal, 2023)	SVM, KNN, DT, RF, MLP + RFE + PCA/t-SNE, 1D/2D CNN, Wav2Vec 2.0, BERT, BETO, RNN, MKL, MLP, SHAP, Chi-square, Extra Trees, Correlation + DT, KNN, RF, Boosting, Feed-forward Neural Network (FNN), KSVM	Selections of features and reductions of data’s dimensionalities, can enhance classifications Applications using PCA and t-SNE on Speech data outperformed languages and multimodal fusions; fusions degrade performances due to temporal misalignments. ExAI identified vocal biomarkers; probability score enabled monitors and DT have high accuracy; SVM application on reduced voice features differentiated PD patients effectively FFNN achieved highest overall classifications in benchmarks with other tested models.	Supported non-invasive voice based PD screening; Highlighted multimodal fusion limits in voice based PD diagnostics Improved clinical interpretability and longitudinal assessments Supported reduced and interpretable PD diagnostics Highlighted remote and cost-effective screening; Enhanced analytic rigors for non-invasive PD detections.	Singular datasets; Only binary classifications; No external validations Fusions are sensitive to temporal scales with under performance of languages Cross-sectional designs. No quantifications of uncertainties or estimates; inadequate comparative analysis.
Italian dataset, voice dataset (Costantini et al., 2023; Mahesh et al., 2024)	KNN, SVM, Naïve Bayes + CFS; CNN, KNN, RF, SVM, XGBoost; Ensemble	ML/DL approaches attained comparable performances; Feature selections assisted in discriminating treated/untreated PD patients from the healthy. RF–XGBoost ensemble combinations performed better than outperformed singular classifiers.	Demonstrated voice comprehension of diseased stages/medication statuses, enhancing robustness of methods in moderate sized datasets.	Moderate sample sizes; language specific datasets Performance dependence on datasets; absence of external validations.
Vowels, Speech (Cesarini et al., 2023; Pragadeeswaran & Kannimuthu, 2024)	GA, NB, RF, MLP, DHW, STFR, AIPB QCNN	Genetic Algorithm (GA) + Naive Bayes (NB) performances were highest; most information from spectral, cepstral and prosodic features; Optimized pipelines improved classifications in comparison to previous models.	Enabled differential diagnosis of PD based voice disorders from others; Demonstrated advanced optimizations in PD related voice diagnostics.	Measured conditions for recording voices in Italian language; High computational complexity; inadequate interpretability.
Wearable motion signals (Babu et al., 2023)	K-means, KNN, SVM, DNN, RNN	High discrimination of hand postures/movement patterns of NDDs by RNNs.	Motor dysfunctions monitored on a continuous basis with reduced costs and in a Non-invasive, manner.	Neural pathological inferences were not direct; absence of confidence intervals/external validations.
Spiral and wave drawings (Saravanan et al., 2023)	CNN (VGG19–INC) with LIME	Hybrid VGG19–INC performed better than pretrained CNNs for classifying PD cases	Detected motor impairments early with comprehensible visual signs.	Absence of confidence intervals, improper dataset sizes, and general constraints.
MRI (Structural/Diffusion) (Islam et al., 2024; Vaillancourt et al., 2025)	Transfer learning CNN (DenseNet169 + ANN), LIME, Linear SVM (AIDP)	DenseNet169 using controls was most accurate in identifications of PD, MSA and PSP instances in multicentered dataset	Demonstrated explainable DL on voluminous MRI datasets.	Absence of external validation and confidence intervals; anatomical interpretations were limited.

## ML/DL techniques used for NDDs

**Figure 7:**
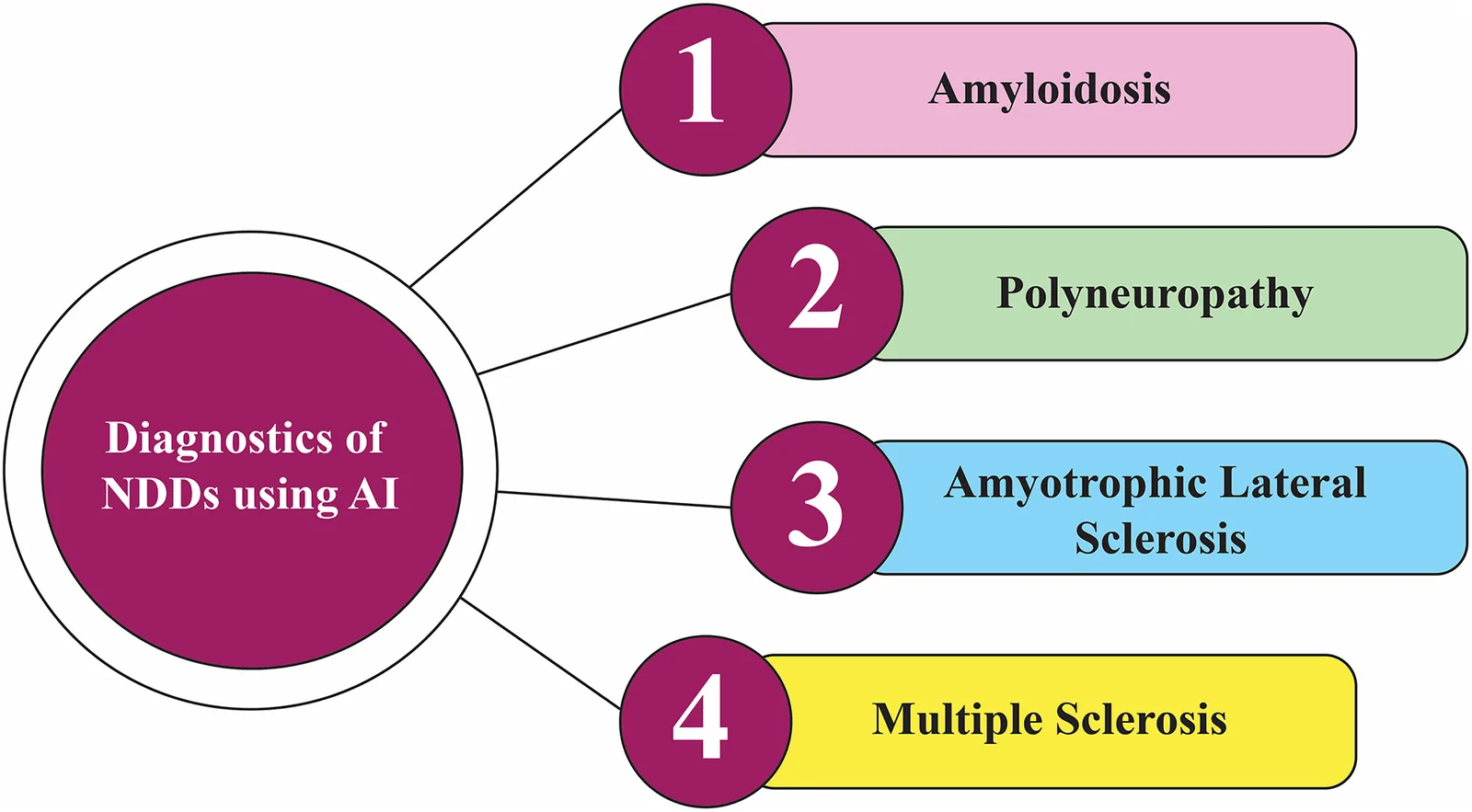
Diagnostics of NDDs using AI.

Chronic NDDs like MS, ALS, and hereditary transthyretin amyloidosis with polyneuropathy (ATTRv) provide significant diagnostic and prognostic issues because of their clinical variations, symptom overlaps and unexpected disease pathways. Recent breakthroughs in AI (refer to Fig. 7), notably ML/DL and NLP, have made it possible to analyze complex clinical and imaging data, giving established diagnostic pathways additional tools for NDDs. DL methods are used for high-dimensional imaging data, NLP for unstructured clinical narratives and traditional ML for organized information.

GRF derived gait features were examined in Vaillancourt et al. (2025) for identifying and classifying HD and ALS using a variety of ML/DL models including one-dimensional CNNs (1D CNN), RF, MLP and ensemble approaches. The study showed gait dynamics as common motor biomarkers for many NDDs where classification performances on all performance metrics showed higher values across disease-*vs*-control tasks. An NLP-based system of Hens, Wyers & Claeys (2023) automatically identified red flag symptoms that might indicate ATTRv polyneuropathy in unstructured EHRs. The algorithm examined interdisciplinary EHRs from 1,015 patients using NLP pipelines trained and optimized on clinical data. The study found 128 patients with three or more predetermined red flags. Sixty nine patients were advised to undergo genetic testing following clinical examinations by experts. The model demonstrated excellent accuracy and raised genetic testing rates by 48.6%, with F1-scores ranging from 0.88–0.98. The work illustrated NLP based detections of rare multi-systemic NDDs from EHR reviews. The work in Gende et al. (2023) examined AD, PD, MS and ET using DL based retinal layer segmentations with optical coherences after transitioning from clinical texts to imaging data. CNNs were trained using various data availability conditions to ascertain impacts of disease specific retinal changes on segmentations. Models training on datasets with numerous NDDs increased their resilience and generalizability. The work demonstrated the importance of disease-induced structural heterogeneity in modeling for guaranteeing better downstream biomarker extractions in neuro-ophthalmology applications. An exemplar-based AI system of Ekmekyapar & Taşcı (2023) integrated traditional ML classifiers with a lightweight DL architecture (MobileNetV2) for MS diagnostics using neuroimaging. The model used Iterative Minimum Redundancy Maximum Relevance (IMRMR) based feature selections and KNN classifications to extract deep features from MRI images which were split into small image patches. The work accurately identified MS instances from two different datasets (Datasets 1 and 2). In addition to imaging-focused approaches, the study in Segura et al. (2023) investigated unstructured EHRs of 250 ALS patients (mean age 64.7 years; 61.6% male) treated at several Spanish hospitals using NLP. The study found 36% of the patients had bulbar-onset ALS while 64% had spinal-onset ALS. The median diagnostic delays from disease onset were 11 months for symptoms like fasciculations, dysphagia, dysarthria and dyspnea. On 2 year median follow-ups on patients: 87.6% had riluzole, gastrostomies were found in 52%, non-invasive ventilations percentage was 64% and tracheostomies identified in; 16.4%. These treatments were substantially more common (all *P* < 0.05) and more likely to occur in bulbar-onset ALS (all *P* < 0.03) than in spinal-onset ALS. The study highlighted diagnostic delays in ALS, clinical feature subtypes and treatments related to diseases. The prediction model of Li et al. (2023) used data of 2,226 participants, aged between 60 to 80 from 2011–2014 National Health and Nutrition Examination (NHANES) Survey for identifying cognitive impairments in older persons. Composite Z-scores were computed using Digit Symbol Substitution Tests, Animal Fluency Tests and CERAD Word Learning and Delayed Recall assessments to evaluate cognitive performances. Initially, thirteen clinical and demographic factors were examined and important predictors selected using Boruta algorithm. Subsequently, RF, SVM, ANN, stochastic gradient boosting (SGB) and generalized linear model (GLM) techniques were used to form models with tenfold cross-validation. The training and test sets were made up of 1,559 and 667 individuals, respectively. Models were built using ages, races, body mass indices (BMI), high-density lipoprotein (HDL) cholesterols, histories of strokes, dietary inflammatory indices, HbA1c, PHQ-9 scores, sleep durations, and albumin levels. The study found cognitive impairments in 384 (17.25%) participants. GLM discriminated best in test sets (AUC = 0.779), indicating that ML models can be reliable instruments for forecasting cognitive deteriorations. These works demonstrate common difficulties in applying AI to the study of neurodegenerative diseases, including uncommon and clinically diverse conditions like MS, ALS, and variant transthyretin amyloidosis (ATTRv). Table 3 summarizes NDDs with their corresponding ML/DL approaches.

**Table 3:** NDDs with their corresponding ML/DL approaches.

Disorder(s)/studies	Data types	AI methods	Key analytic insights
ATTRv (Hens, Wyers & Claeys, 2023)	EHR	NLP (NER, concept extraction)	Diversity of training data is important for DL generalization.
AD, PD, MS, ET (Gende et al., 2023; Ekmekyapar & Taşcı, 2023)	OCT, MRI images	CNN, MobileNetV2, IMrMr, KNN	Excellent results with regulated datasets; external verification is required. Analysis of disease trajectories at the population level is made possible using NLP.
ALS, Cognitive impairment (Segura et al., 2023; Li et al., 2023)	HER, Survey data	NLP, GLM, RF, SVM, ANN, SGB	In structured data, simpler models could perform better than sophisticated AI. Although NLP enhances rare illness screening, clinical validation is necessary.

## Results and Discussions

This section discusses the issues and challenges of ML/DL applications for NDDs from multiple angles, along with probable solutions. The suggestions are intended to help researchers and professionals investigate AI’s potential for NDD diagnosis. They are often characterized by neuronal losses or degeneration and lead to a variety of neuropsychiatric problems. Their extremely complex pathophysiology, which encompasses several pathogenic mechanisms in both their initiation and development, results in a limited survival rate. HD poses serious difficulties for those with the illness as well as their family. Cases of Relapse-Remitting MS (RRMS) are characterized by recurrent flare-ups or relapses followed by partial or complete recoveries. The hallmarks of primary progressive MS (PPMS) are devoid of noticeable relapses or remissions (Klineova & Lublin, 2018). Secondary progressive MS (SPMS) begins as RRMS before deteriorating into symptom stages. Individuals with NDDs can be made to lead a better quality of life by creating novel therapies and comprehending reasons underlying these diseases (Eva et al., 2023). Immunotherapy and medications have increased options for treating patients with MS (Dargahi et al., 2017). Medications for MS include immunosuppressants like mitoxantrone and natalizumab, as well as beta-interferon and copolymer 1. AD is the primary cause of dementia in the elderly (Lamptey et al., 2022). Drugs like Memantine and cholinesterase inhibitors effectively reduce symptoms and slow down cognitive declines in AD. Advanced imaging techniques and cerebrospinal fluid (CSF) investigations may also be used in some circumstances and treatment strategies such as cognitive training and occupational therapies may also prove to be beneficial (Hogan et al., 2008). Progressive decreases in motor functions are hallmark of PD which develops gradually, but the intensity and symptoms vary between patients. The non-motor symptoms include mood swings, cognitive declines and sleeping difficulties. People experience issues in swallowing, vocal communications and cognitive abilities in later stages (Mack & Marsh, 2017). Regular physical exercise is said to be helpful for effectively controlling symptoms and preserving general wellbeing, particularly for activities that increase flexibility and balance. PD patients can live better lives when detected early for appropriate interventions (Jankovic & Aguilar, 2008). ALS onset results in deterioration of Motor neurons, the nerve cells that control voluntary muscular contractions (Zarei et al., 2015). Breathing problems, speech and swallowing difficulty, muscular weakness and muscle atrophy are typical symptoms of this disorder. It can be categorized into two groups: familial ALS and sporadic ALS (Masrori & Van Damme, 2020). The efficiency of muscles and motor neurons is frequently assessed using electromyography (EMG) and nerve conduction studies. By lessening the damage done to motor neurons, the FDA-approved medication riluzole can slow the progression of ALS (Miller et al., 2002). An FDA-appoved adjuvant medication called edaravone is used to treat ALS and can slow the progressive decline of physical ability (Neupane et al., 2023). The findings of this study in diagnostics of NDDs are listed below.

### Advantages of using ML/DL for diagnosing NDDs

- The statistical characteristics of transcriptomic data in place of predictive performance can drive methodological decisions in diagnostics. ANNs can model complicated expression patterns, LASSO can identify sparse and interpretable gene selections, while RF can be used to rank nonlinear features.
- ML frameworks and NLP-driven DL models aid in risk stratifications when used in the proper analytical contexts and structured-data in the development of cognitive impairments.
- Gait dynamics are promising complementary biomarkers for early detection of NDDs. Task-specific gait tests can improve sensitivity to small impairments, especially when they impose higher cognitive demands.
- Studies demonstrate how AI may be used in biosensor technologies for NDDs in two complementary ways: by combining several low-abundance biomarkers to enhance diagnostic performances and resolving complicated signal patterns originating from diverse biomolecular states. Biosensor technology driven by AI illustrates that complex spectroscopic signatures of protein aggregations can be decoded by the use of AI based sensors, and plasma biomarkers can assist in disease screening.
- Voice-based AI systems are more appropriately regarded as supplemental instruments that can facilitate illness monitoring, early screening, and customized evaluation than as stand-alone diagnostic substitutes. DL optimization based approaches in Voice-based AI applications provide complementary benefits for greater computational complexity where feature-driven ML pipelines using feature selections/dimensionality reductions perform better.
- AI model performances for sensor based devices are dependent on selections of modalities, feature representations, candidate algorithms, and validations. Multimodal frameworks that combine behavioral and sensor-based data with neuroimaging markers should be prioritized. AI model selections in light of data structure and disease biology.
- Large MRI datasets may be analyzed in a scalable and automated manner using DL-based techniques. Biologically based feature engineering in conjunction with traditional ML techniques produces more statistically sound and interpretable models.

### Limitations of using ML/DL for diagnosing NDDs

- Despite promising diagnostic potentials, ML/DL techniques are limited by small dataset sizes, dependence on reviewing data, heterogeneity of symptoms, platforms, and absence of longitudinal validations. They are very sensitive to data configurations while training and structural differences of diseases.
- NLP techniques face limitations from EHRs in comprehensiveness, uniformity, and quality of clinical annotations. Furthermore, many described models are based on internal cross-validation rather than external or prospective validations, which limits their immediate clinical generalizability.
- Outcomes of AI models for NDDs are based on the algorithm’s performance mainly, but additionally, data alignments, methodology, clinical contexts, and involvement of clinicians are also important factors of interpretability.
- Studies about Gait and spatiotemporal biomarkers have limited comparisons due to variations of methods/modalities, inadequate measures of uncertainties.
- Diagnostics with structural images use datasets without external validations or confidence intervals or performance parameters unsupportive of longitudinal analysis.
- In Biosensor technologies, the structures of data and specific analytical problems being addressed need to complement method selections rather than being applied as generic enhancements.
- AI Voice diagnostics are hurdled by difficulties in temporal alignments, cross-section designs, and multimodal depictions in learning, which dilute voice discriminative power. They are also hampered by small datasets, voice sources, architecture of models and validation procedures.
- The use of accuracy point estimates without confidence ranges and restricted benchmarking restrict clinical interpretabilities and comparisons of sensor based AI techniques.

## Conclusion

ML/DL approaches have emerged as powerful analytical frameworks in the study of NDDs, enabling the methodical examination of intricate, high-dimensional data that traditional statistical methods cannot handle. AI-based algorithms have continuously shown promise in discovering diagnostically relevant patterns spanning all NDDs for a wide range of data, including transcriptomics, biosensors, neuroimages, speeches, gait and EHRs.

### Future research directions

Future studies should compare ML/DL techniques against well-established classical baselines under controlled circumstances, focusing more on therapeutic relevance, stability and computing efficiency than just novelty. The wide range of reported accuracies among studies suggests that performance metrics need to be interpreted in the context of their particular experimental environments. Future studies should prioritize large, multi-center longitudinal data sets, standard assessment techniques, explainable models that link clinical reasoning with computational outputs, and uncertainty-aware reporting. Imaging researchers must integrate scientific rigor and scalability, with an emphasis on consistent acquisitions, external validations, and transparent reporting of uncertainties to enable appropriate clinical integration. The use of DL in structural imaging requires improved class balances and comprehensible clinical decision assistance. Standardizing analytical procedures and openly disclosing uncertainty are essential for future clinical translation. Future studies should also concentrate on prospective validation in therapeutically significant groups, transparent presentation of analytical results, including uncertainty estimates, and harmonized multi-site datasets.

## Additional Information and Declarations

### Data Availability

The following information was supplied regarding data availability:

Gitup Link: https://github.com/SMohanraj4036/Systematic-Review-of-Neurodegenerative-Diseases

Zenodo Link: DOI 10.5281/zenodo.18898570

This manuscript is a systematic literature review. All data discussed in this article are obtained from previously published studies and are available from the corresponding references cited within the manuscript.
